# Paediatric UK demyelinating disease longitudinal study (PUDDLS)

**DOI:** 10.1186/1471-2431-11-68

**Published:** 2011-07-28

**Authors:** Michael Absoud, Carole Cummins, Wui K Chong, Christian De Goede, Katharine Foster, Roxanna Gunny, Cheryl Hemingway, Philip Jardine, Rachel Kneen, Marcus Likeman, Ming J Lim, Mike Pike, Naomi Sibtain, William P Whitehouse, Evangeline Wassmer

**Affiliations:** 1 School of Health & Population Sciences, University of Birmingham, Vincent Drive, Birmingham, B15 2TT, UK; 2 Department of Neurology, Birmingham Children's Hospital, Steelhouse Lane, Birmingham, B4 6NH, UK; 3 Department of Neuroradiology, Great Ormond Street Hospital for Children, Great Ormond Street, London, WC1N 3JH, UK; 4 Department of Paediatric Neurology, Royal Preston Hospital, Sharoe Green Lane North, Lancashire PR2 9HT, UK; 5 Department of Neuroradiology, Birmingham Children's Hospital, Steelhouse Lane, Birmingham, B4 6NH, UK; 6 Department of Neurology, Great Ormond Street Hospital for Children, Great Ormond Street, London, WC1N 3JH, UK; 7 Department of Neurology, Bristol Royal Hospital for Children, Upper Maudlin Street, Bristol, BS2 8BJ, UK; 8 Department of Neurology, Alder Hey Children's NHS Foundation Trust, Eaton Road, Liverpool, L12 2AP, UK; 9 Department of Neuroradiology, Bristol Royal Hospital for Children, Upper Maudlin Street, Bristol, BS2 8BJ, UK; 10 Department of Neurology, The Evelina Children's Hospital at Guy's & St Thomas' NHS Trust, Great Maze Pond, London, SE1 9RT, UK; 11 Department of Neurology, Oxford Children's Hospital, Headley Way, Oxford, OX3 9DU, UK; 12 Department of Neuroradiology, King's College Hospital NHS Trust, Denmark Hill, London, SE5 9RS, UK; 13 Academic Division of Child Health, Queen's Medical Centre, Nottingham, NG7 2UH, UK

## Abstract

**Background:**

There is evidence that at least 5% of Multiple sclerosis (MS) cases manifest in childhood. Children with MS present with a demyelinating episode involving single or multiple symptoms prior to developing a second event (usually within two years) to then meet criteria for diagnosis. There is evidence from adult cohorts that the incidence and sex ratios of MS are changing and that children of immigrants have a higher risk for developing MS. A paediatric population should reflect the vanguard of such changes and may reflect trends yet to be observed in adult cohorts. Studying a paediatric population from the first demyelinating event will allow us to test these hypotheses, and may offer further valuable insights into the genetic and environmental interactions in the pathogenesis of MS.

**Methods/Design:**

The Paediatric UK Demyelinating Disease Longitudinal Study (PUDDLS) is a prospective longitudinal observational study which aims to determine the natural history, predictors and outcomes of childhood CNS inflammatory demyelinating diseases. PUDDLS will involve centres in the UK, and will establish a cohort of children affected with a first CNS inflammatory demyelinating event for long-term follow up by recruiting for approximately 5 years. PUDDLS will also establish a biological sample archive (CSF, serum, and DNA), allowing future hypothesis driven research. For example, the future discovery of a biomarker will allow validation within this dataset for the evaluation of novel biomarkers. Patients will also be requested to consent to be contacted in the future. A secondary aim is to collaborate internationally with the International Paediatric Multiple Sclerosis Study Group when future collaborative studies are proposed, whilst sharing a minimal anonymised dataset. PUDDLS is the second of two jointly funded studies. The first (UCID-SS) is an epidemiological surveillance study that already received ethical approvals, and started on the 1st September 2009. There is no direct patient involvement, and UCID-SS aims to determine the UK and Ireland incidence of CNS inflammatory demyelinating disorders in children under 16 years.

**Discussion:**

A paediatric population should reflect the vanguard of MS epidemiological changes and may reflect trends yet to be observed in adult MS cohorts. The restricted window between clinical expression of disease and exposure to environmental factors in children offers a unique research opportunity. Studying a paediatric population from the first demyelinating event will allow us to investigate the changing epidemiology of MS, and may offer further valuable insights into the genetic and environmental interactions in the pathogenesis of MS.

## Background

### Introduction

CNS inflammatory demyelinating diseases (CIDD) are rare childhood disorders but may culminate in physical and cognitive disability or ultimately be diagnosed as Multiple Sclerosis (MS). MS is a chronic inflammatory demyelinating disease of the CNS characterized by myelin loss, axonal degeneration and, often, progressive neurological dysfunction, that is usually relapsing remitting at onset. The incidence of childhood CIDD is unknown, and the UK and Ireland Childhood Inflammatory Demyelination Surveillance Study (UCID-SS) is already underway to determine this. At the first presentation of a CNS inflammatory demyelinating event, children are diagnosed with either an acute disseminated encephalomyelitis (ADEM), optic neuritis, transverse myelitis, or another clinically isolated episode (CIS). Children who present with a CNS inflammatory demyelinating episode involving single or multiple symptoms may later present with a second event (majority usually within two years) to then meet criteria for MS diagnosis [1]. There is evidence that at least 5% of MS cases manifest in childhood [2]. It is not clear at the first onset of symptoms which children will go on to develop MS.

### Prospective studies are required

The available literature on paediatric MS, is primarily limited to smaller case series and larger retrospective reviews of established adult MS populations [3-16]. The International Paediatric MS Study Group, (http://www.ipmssg.org) have recently published consensus definitions of paediatric CNS inflammatory demyelinating disorders and MS to help facilitate uniformity in future research [17]. Evidence for differences in natural history of childhood onset MS [18] are also primarily derived from retrospective cohorts, as are studies linking environmental factors (EBV infections or Vitamin D deficiency) with possible MS development [19]. The risk of relapse in children and hence the risk of developing MS may be geographically different [1]. There is hence an urgent need for prospective population based studies to further understand clinical, radiological, and pathobiological features as well as outcome of childhood onset inflammatory demyelinating disease.

### Paediatric cohort may reveal new trends

Various new trends in adult MS have been reported (reviewed in ref [20]), which include geographic rising rate of MS, and a rising female to male sex ratio by year of birth [21]. There is evidence that Indian and Pakistani immigrants who entered England younger than 15 had a higher risk of developing MS than those that entered after this age [22]. The vanguard of any change in trends in the epidemiology of MS is likely to be evident in a paediatric population. Genetic epidemiology to date supports the dual role of "nature and nurture" in MS pathobiology [20]. As such paediatric cohorts can also offer further valuable insights into the genetic and environmental interactions in the pathogenesis of MS. The corner stone of cohorts that are likely to be able to address complex disorders such as MS, depend on the availability of biological sample with comprehensive databases with clinical information.

### New criteria for diagnosis for paediatric MS require evaluation

At first presentation of CIDD, children are diagnosed with optic neuritis, transverse myelitis, other clinically isolated episodes (CIS) or acute disseminated encephalomyelitis (ADEM). The newly proposed consensus definitions [17] for CIDD are operational and require evaluation in prospectively followed up cohorts. For isolated monophasic syndromes, normal MRI and cerebrospinal fluid (CSF) are thought to predict a very low risk for development of MS in adults [23]. However the same may not be true for children. In addition the differential diagnosis for childhood demyelinating disease and MS (documented in exclusion criteria) is much wider than that in adults. Furthermore, MRI criteria used in adults to diagnose MS e.g. MRI McDonald criteria may not be valid in children [24-26]. The utilisation of revised McDonald criteria [27] and others including the KIDMUS MRI criteria [10] for paediatric MS need to be validated in new cohorts.

McDonald et al. [27] criteria:

Three of: 1) ≥ 9 white matter lesions or 1 gadolinium enhancing lesion, 2) ≥ 3 periventricular lesions, 3) 1 juxtacortical lesion, 4) an infratentorial lesion.

KIDMUS [10] criteria:

All of: ≥ 1 lesion perpendicular to long axis of corpus callosum; sole presence of well defined lesions

### No available biomarker for MS

In line with adult based studies [28], there is no currently available biomarker that fulfils the criteria of a surrogate endpoint in MS in children. Current routine tests including CSF oligoclonal bands, lack sensitivity and specificity in individuals presenting with a first CNS inflammatory demyelinating event. A precondition for the identification of biomarkers is a well described and prospectively followed clinical cohort and the availability of stored tissue linked to relevant clinical information.

### Early diagnosis allows for early treatment

For relapses or attacks of MS, corticosteroids are the mainstay of treatment. Early initiation of "disease modifying agents", such as beta interferon and glatiramer acetate appears to be of benefit in adults. Thus, it is more critical than ever to accurately diagnose MS as early as possible. However, very little is published on the use of interferon in children [29-33]. The determination of the longitudinal outcome of the first CNS inflammatory demyelinating episode in children is an essential pre-requisite for the design of any prevention or treatment trials. Prediction of outcome at an early stage is critical to quantify the risk to benefit ratio of any intervention.

### The need to know the extent of disease burden

Furthermore, identifying the natural history and outcomes of paediatric MS in the UK has major implications when planning service provisions, where there is evolving evidence of lack of awareness, and patchy service provision. At the Paediatric MS meeting, London November 2007 (attended by families and multidisciplinary professionals) there was a call for a better understanding of long-term outcomes including quality of life, cognitive, adaptive behavioural and functional skills, neuropsychiatric symptoms, and treatment effects for children presenting with CNS inflammatory demyelination.

### Context of study in relation to current practice

Children with CNS inflammatory demyelination are most often routinely seen by a Paediatric Neurologist as part of clinical care in order to provide expert advice and management. In line with guidelines by the International Paediatric MS Group, demyelination clinics have been set up in several Tertiary Centres by paediatric neurologists with an interest in these conditions. This led to the UK and Ireland Paediatric CNS Inflammatory Demyelination Working Group being set up, providing a steering committee for future collaborative studies, networking, future direction, writing clinical guidelines and future grant applications. As a result, the majority of these children are seen in these specialist centres where they will be recruited.

### Study aims and objectives

**Figure 1:**
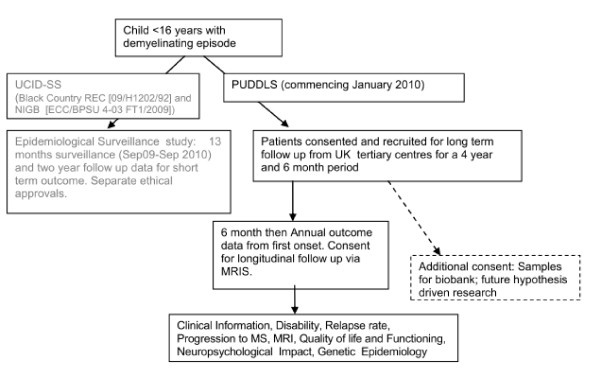
**PUDDLS Flowchart with link to epidemiological study**.

PUDDLS aims to establish if prognostic factors for relapse of CNS inflammatory demyelination (and hence risk of progression to MS) can be identified for UK children (Figure 1). PUDDLS is a prospective UK based study with the following objectives to:

1. Identify features (clinical, epidemiological, imaging, pathophysiolgical tests such as oligoclonal bands) that may predict which children are more likely to relapse and develop MS, whilst assessing the validity and utility of the new consensus definitions for childhood CNS inflammatory demyelination and of proposed MRI criteria.

2. Establish outcomes for children, for an initial period of up to five years depending on when the case was recruited (minimum 6 months follow up).

3. Describe disease modifying therapy usage, efficacy, and adverse effects.

4. Provide a platform for future a) identification of biological markers in MS, b) genetic studies and c) hypothesis driven biological studies; by storing blood and CSF samples already taken at the time of first demyelinating event (currently in 7 participating centres).

5. Ensure that long term outcomes of childhood demyelinating disease are as complete as possible by flagging patients with their consent with the NHS Information Centre [Medical Research Information Service (MRIS)] which will allow them to be traced in future via their G.P.s (currently in 7 selected centres).

### Specific Hypotheses

We wish to test the following *hypotheses *(as suggested by the IPMSSG [17]):

(i) Children who present with CIS are more likely to develop MS than children who present with ADEM.

(ii) ADEM Children with prolonged steroid- dependent events (≥ 3 months) are more likely to develop MS than children with rapid recovery ADEM.

(iii) Children with multiphasic ADEM compared to children with recurrent ADEM are more likely to develop MS.

(iv) Children with recurrent ADEM are more likely to develop MS than children with monophasic ADEM.

(v) MRI criteria (McDonald, KIDMUS) can be validated to distinguish between ADEM, ADEM variants, CIS, and MS.

(vi) Identified future biomarkers with suggested prognostic significance, can be tested against PUDDLS outcomes.

(vii) Early treatment with disease modifying therapy in relapsing CNS Inflammatory Demyelination, reduces relapse rate and slows neurological disability progress.

(viii) Environmental risk factors can be identified for children with first episode CNS inflammatory demyelination and MS.

New hypotheses will be added if suggested by publications by other centres prior to data analysis.

## Methods/design

### Case Definitions and Inclusion Criteria

Children (younger than 16 years) experiencing first episode of clinical neurological events consistent with site specific inflammatory CNS demyelination and except in Optic Neuritis confirmed with white matter changes on MRI (table 1).

**Table 1:** PUDDLS Inclusion Criteria

Condition	Inclusion Criteria
Acute Disseminated Encephalo-myelitis (ADEM)	A polysymptomatic clinical event with acute/subacute onset that must include encephalopathy (behavioural change or altered consciousness). (2) MRI Brain shows f multifocal lesions, predominantly involving white matter. • Relapsing ADEM: symptoms or signs within 3 months of initial onset of ADEM. IF a new event occurs ≥ 3 months later and ≥ 1 month after completing steroid treatment, it is defined as: • Recurrent ADEM: recurrence of initial symptoms without involvement of new clinical areas. • Multiphasic ADEM: New event, but involving new anatomical areas of the CNS.

Clinically Isolated Syndrome (CIS)	A first acute-clinical episode of CNS symptoms which may either be monofocal or multifocal, but does not include encephalopathy (except in brainstem syndromes). The MRI will show area of white matter demyelination. These include: ***Transverse myelitis***: weakness and/or numbness of both legs +/- arms, usually with maximal deficits 1 week after symptom onset supported by demyelination on MRI spine. ***Brainstem***, ***cerebellar***, and/or ***hemispheric***dysfunction, supported by demyelination on MRI.

Optic Neuritis (ON)	Acute or subacute loss of vision and ≥ 1 of: relative afferent pupillary defect (unilateral cases), visual field deficit or scotoma, impaired colour vision, optic disc oedema, or abnormal visual evoked potentials. MRI is not necessary for diagnosis.

Neuro-myelitis Optica (NMO)	Three of the following four criteria: i. Optic neuritis ii. Acute myelitis iii. Spinal MRI lesion extends over three or more segments iv. NMO antibody testing is positive.

### Exclusion criteria

1. Leukodystrophies (e.g., metachromatic leukodystrophy, adrenoleukodystrophy) or mitochondrial disease.

2. Proven CNS infection (e.g. bacterial meningitis, herpes simplex encephalitis, Lyme disease, HIV).

3. Radiation/chemotherapy associated white matter damage.

4. Condition fulfilling criteria for CNS connective tissue disease e.g. lupus, vasculitis. The sole presence of antibodies associated with CNS connective tissue diseases is not sufficient for exclusion.

### Relapses, MS, and Progression

A relapse is a new occurrence of neurological symptoms that lasted > 24 hours and stabilized or resolved either partially or completely. MS is defined in table 2 below. Progression of MS is a continuous worsening of symptoms and signs for > 6 months, ± superimposed relapses, either primarily (at onset of disease) or secondarily (after first relapse).

**Table 2:** Multiple Sclerosis definition

**Multiple Sclerosis (MS)**	- Two or more non ADEM episodes of CNS demyelination separated in time (4 weeks) and space. For children aged > 10 years: - Dissemination in space can be met if: MRI shows three of: 1) ≥ 9 white matter lesions or 1 gadolinium enhancing lesion, 2) ≥ 3 periventricular lesions, 3) One juxtacortical lesion, 4) an infratentorial lesion. OR abnormal CSF (oligoclonal bands or elevated IgG index) with 2 lesions on the MRI (one in the brain). - Dissemination in time can be met if: MRI shows new T2 or gadolinium enhancing lesions developing ≥ 3 months after initial event.

### Design

**Figure 2:**
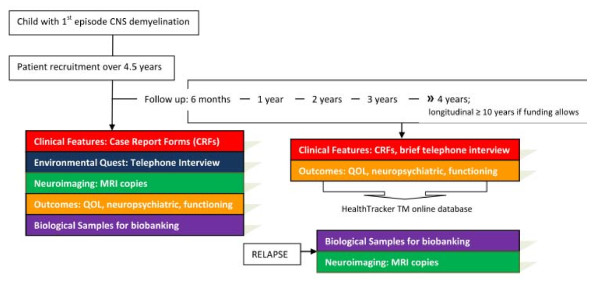
**The 5 major study themes are represented in flow the Figure below**.

**Figure 3:**
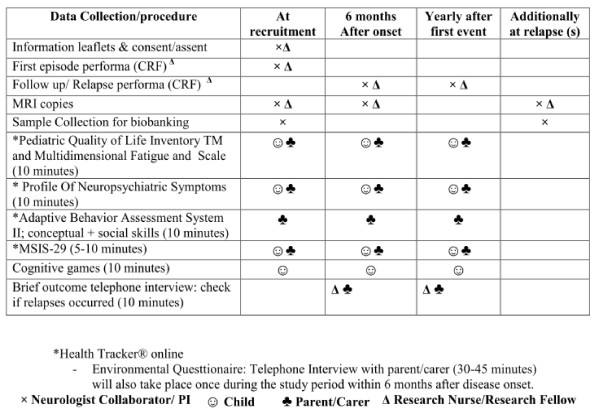
**Data and Outcome Collection Schedule Summary**.

PUDDLS ia a observational cohort study with sample biobanking which will characterize the spectrum of CNS demyelinating syndromes, their outcomes, and possible progression to MS (Figure 2). Children will be recruited over a 4 year and 6 month study period and followed up. At recruitment an initial Case Report Form (CRF) will be completed by the PI/Research Nurse/Research Fellow. MRI copies and biological samples will be archived. An environmenal questtionaire will be administered by telephone interview within three months of disease onset. Patient reported and proxy outcome questtionaires will be completed online at onset and at specified time intervals (Figure 3). At six months and one year from onset, and then at yearly intervals, a follow-up CRF will be completed in clinic. A telephone interview will also be conducted at the same frequency to ask if the patient has had a clinical relapse or hospital admission and to provide an opportunity to ask questions. Any significant concerns will be referred back to the GP or clinician in charge.

### Patient recruitment and consent

Based on an expected incidence of 1-2/100,000 children, each Centre is expected to recruit approximately 10-15 cases/year over the 5 year length of the study, with a minimum of 6 months follow up. Children with CNS Inflammatory Demyelination are seen by Paediatric Neurologists with an interest in CNS Demyelination to provide expertise in diagnosis and management. Patients usually present to Neurologists' attention via the following routes:

- Presentation directly to Tertiary Centre.

- Referral from Paediatric DGH Consultant for admission or an outpatients opinion.

- MRI scans referred to weekly Tertiary Centre Neuroradiology meeting for an opinion (permission to approach the clinician for referral will be obtained).

- Shared care or outreach clinic.

Patients will be ascertained via these available methods, and given or sent information leaflets before being consented (minimum 24 hours) to take part in the study. A website (http://www.childdemyelination.org.uk) is set up to provide newsletters and information regarding study progress, and up to date information on management directed at Paediatricians. If the patient or carer requests referral, normal clinical follow up patterns of care will be followed.

Currently in 7 centres, PIs will obtain consent from families presenting to their centres. The Paediatric Research Nurse at each site (0.1 WTE at each site, except lead Centre [Birmingham] to have 0.3 WTE) or the Research Fellow (Dr M Absoud) may also approach families directly to obtain consent. Appropriate agreements and contracts will be in place for the Research Fellow and Nurse as recommended by the National Institute for Health Research. Patients will be asked to consent to:

- Taking part in the study and l**ongitudinal follow up **via The National Health Service Information Centre [Medical Research Information Service (MRIS)]

- Storage of **Samples **(CSF, DNA, serum)

- Obtaining **MRI **copies and information from Hospital notes

- Sharing an anonymised dataset with the International Paediatric Study MS Group.

### Data Collection and Outcome assessment

Data collection will take place at recruitment, 6 months after initial event, annually after initial event, and during clinical relapses (see Figure 3). Clinical follow ups for patients affected with CNS inflammatory demyelinating diseases will be part of clinical care, and no extra hospital visits will be required. Children and parents/carers will be able to fill in questionnaires from a secure online programme (Healthtracker TM). Access to a computer with privacy will be made accessible if needed during clinic visits. The Research Nurse will make contact with the family at the designated follow up times, to ensure they understand the research, ask about any relapses or investigations the child might have had, and discuss further queries.

*The primary outcome measures *will be (1) if the patient developed a clinical relapse, and (2) a diagnosis MS. Secondary outcome measures will include: Patient reported and proxy Quality of life, disease impact, neuropsychiatric & fatigue symptoms, conceptual & social adaptive behavioural functioning skills, MRI criteria, and objective EDSS disability scores (ie. EDSS > 5.5, patient unable to walk without aid).

### Case Report Forms (CRFs)

The CRFs have been adapted with permission after consultation with the International Group. The initial CRF (first episode) will be completed by the PI, the Research Fellow, or the Research Nurse with assistance from the PI. A condition specific targeted neurological history (using a check box questionnaire) would take place at the time of first demyelinating event. The event would be classified as either ADEM, CIS, or MS (see case definition). Data collected can broadly be categorised to:

a. Basic demographic data (ethnicity, place of birth, current residence).

b. Family history of MS or autoimmune disease.

c. Demyelinating symptoms and signs (using tick box table).

d. Presence of behavioural disturbance or encephalopathy (to distinguish ADEM from CIS) at time of demyelinating event.

e. Information which might predict the subsequent development of recurrent ADEM, multiphasic ADEM, or MS, like details of the anatomic distribution (optic nerve or spinal cord, monofocal or multifocal distribution), CSF findings, serological and neuroimaging results.

f. Treatment (eg, steroids, immunoglobulins, intensive care) given.

g. Information as to whether the risk of future MS was given to the family.

h. Neurological examination, Kurtze EDSS scales, and functional status.

For **6 **month and **annual **outcome data, follow up information will be obtained on neurological function and further demyelinating events (if these have occurred). We aim to collect 90% data within a window period of one month on either side of the designated follow up periods.

### Healthtracker^®^; patient self report and proxy outcomes

PUDDLS will use Healthtracker^® ^which is a sophisticated, user-friendly, UK, and web-based assessment system launched with Department of Health support in 2008. It comprises a suite of questionnaires and test games for children and their carers, and not for investigator use. These allow accurate measures of change across a wide range of symptoms, side-effects, psychological functions and quality of life. Assessments can be home-based for convenience and lower costs. This helps circumvent the many practical difficulties associated with long-term assessment of chronic diseases.

#### Health related Quality of Life (QOL)

- Generic QOL: Pediatric Quality of Life Inventory TM;

#### Generates physical, psychological, and total score

○ Self report for ages 5-25 years.

○ Parent report for ages 2-25 years.

- Disease specific QOL: Multiple Sclerosis Impact Scale-29 version 2 (MSIS-29 v2).

#### Generates physical, and psychological scores

○ Self report for children 5 years -adulthood.

○ Parent report.

#### Fatigue

- Pediatric Quality of Life Inventory TM; Multidimensional Fatigue Scale

○ Self report for children 5-25 years

○ Parent report; for children 2- 25 years

#### Neuropsychiatric Symptoms

- Profile Of Neuropsychiatric Symptoms (PONS)

○ Self report for children 5-16 years

○ Parent report

#### Functioning; Adaptive behavioural skills

○ Adaptive Behaviour Assessment System II (ABAS II).

- Parent report for children 5 years -adulthood. Two out of the three domains will be used:

(i) Conceptual skills: communication, functional academics, and self-direction skills areas

(ii) Social skills: Social, and leisure skills areas

#### Cognitive

○ Two Healthtracker online games played by children aged 5-16 years that aim to measure memory, attention and concentration. These are embedded in between questionnaires.

○ Online D2 test game for participants aged 9 years- 60 years. The D2 Test measures processing speed and quality of performance of individuals, allowing for a neuropsychological estimation of individual attention and concentration performance.

### Environmental Questionnaire

One off telephone interview (30-45 minutes) with parent/carer to be conducted during study period within three months of disease onset if direct hospital contact whilst inpatient has not been achieved. Only once further funding is gained, case controls for administering only the environmental questionnaire will be ascertained via a 'buddy system' to the child who is age and sex matched. No other involvement of controls will be required.

### MRI criteria

Routine patient MRIs will be collected in order to apply proposed criteria (such as McDonald, KIDMUS) that the study team can validate against PUDDLS outcomes. Data will be collected using a standardised data collection performa with agreed definitions designed by Neuro-radiology collaborators (available on request). MRIs will be randomly evaluated by one of four Neuroradiologists blinded to presentation and clinical features. Inter-rater reliability will be assessed.

### Biobank sample collection

#### Samples and Procedures

Additional informed consent will be obtained to store sera, DNA, and CSF collected during routine clinical care at first onset of a CNS demyelinating event, and then at future hospital visits if clinical relapses occur. This will be done in accordance with the Human Tissue Act (HTA, 2004). Currently designated laboratories in each of the seven establishments hold appropriate HTA licences and would be responsible for storage:

(http://www.hta.gov.uk/licensingandinspections/listoflicensedestablishments.cfm)

1. Alder Hey Children's NHS Foundation Trust

2. Birmingham Children's Hospital

3. Frenchay Hospital

4. Great Ormond Street Hospital

5. Guy's & St Thomas' Hospitals, Cellular Pathology

6. John Radcliffe Hospital

7. Nottingham University Hospitals NHS Trust

Sample collection, transport and storage will be undertaken using standard SOPs and be based at collaborating centres as appropriate.

There will be no additional venepuncture or lumbar puncture procedures for the child beyond those required for routine clinical care. Informed consent will be required, for extra volumes to be collected during these procedures for the study. Residues of CSF and blood already taken as part of the clinical management of the patient, will also be used for future research investigations and to supplement the specimen archive. For each patient at least one CSF sample and two blood samples will be required. Clinicians will be encouraged to follow existing guidelines for the investigation of CNS demyelinating disorders (17,18) and as updated in the future.

### Confidentiality

Samples will be stored pseudoanonymised in HTA approved labs, and linked with the clinical data by the unique PUDDLS number. Any information that is likely to identify participants (such as name, address, date of birth) will be removed from samples. Personal identifiers are kept separately under strict control with restricted access in secured cabinets, at the Institute of Child Health, Birmingham Children's Hospital in a secure locked area. Access to data is limited to the Chief and Principal Investigators. The study administrator at Birmingham Children's Hospital will ensure pseudo-anonymous tracking of the samples, solely for the purposes of linkage once they are collected as informed by the Principal Investigator. The location, nature and date of samples will be recorded on the database.

### Future research

Separate funding will be sought for the biological/genetic sample studies and a further five year longitudinal follow up, once sufficient samples and clinical data has been collected. The details of the studies are beyond the scope of this protocol but we have identified future potential studies of sufficient scientific validity to ethically justify the storage of human samples. Collaborations with international experts and leaders in the field will maximise yield of investigations, and ensure a thorough review process:

The PUDDLS Biological Studies Steering Group will consider any future studies, and follow the agreed guidelines for study selection (***available on request***). Once studies are deemed of sufficient quality, and depending on the strand of study the group will collaborate with the experts in their fields and with the International Paediatric Multiple Sclerosis Study Group (IPMSSG).

### Expected Numbers

Based on an expected incidence of 1-2/100,00 children, the expected recruitment over the initial 5 year study period is 400-450 cases (12-13/year in every Centre).

### Statistics and data analysis

Descriptive statistics (with confidence intervals where appropriate) will be used to summarise the key components of the database. Univariate associations between potential prognostics factors and potential confounders and with MS diagnosis or other CNS demyelinating episodes; outcomes. These associations will be further explored in logistic regression models that will allow for control of confounding factors. Factors associated with early occurrence of second episodes will also be explored and Cox models will be used if the proportional hazards assumption is met. Life table analysis methods will be used to calculate primary outcome time points. SPSS (v.17.0) statistical software will be used for data analysis.

### Research and development procedures

Research & Development (R&D) approval from each participating NHS Trust will be obtained prior to recruitment. Principal investigators will follow rules of data protection ethics and confidentiality.

#### Longer term follow up

To ensure that long term outcomes of childhood demyelinating disease can be identified by flagging patients with their consent via the NHS Information Centre (MRIS) and NHS Central Register which will allow them to be traced in the future via their G.P.s. We will consent patients for long term follow-up.

### Data Handling and Integrity

• Each patient enrolled will be assigned a unique PUDDLS study identification number. This number will be used in all future correspondence between the study centre and principal investigators.

• Principal investigators will be responsible for the security of data at each local site according to their employing authorities' rules and regulations. Investigators will ensure that the study is conducted and data are generated, documented and reported in compliance with the protocol, and applicable regulatory requirements.

• Patient identifiable information (name and address) will be sent to the study office on only one occasion (initial case report form) by secure fax. The original signed consent form to participate in the study will be kept in the patient's medical records unless required otherwise by local regulations, and a copy kept securely in the site file.

• At the central study office (ICH, Birmingham Children's Hospital), all patient identifiable study data will be stored in locked filing cabinets. Identifiable patient data will be separated from the clinical data and linked with a unique study code. Clinical data used for research may also include patient identifiable data, e.g. date of birth, sex, that are also important for the data analysis, linkage during follow up, and to answer the research questions. These cannot be removed from the questionnaire and will be stored for 30 years in secure archives (MRC guidelines). Only pseudo-anonymised clinical data only will be entered into the electronic study database for analysis. Case report forms will be kept in the site file in a secured locked cabinet, and a copy will be sent to the Central study office at the Institute of Child Health, Birmingham Children's Hospital.

• Database Information

(i) Clinical Information

The research nurse, data administrator, and/or the research fellow will enter and maintain the clinical information for all study participants from within the Institute of Child Health (ICH), Birmingham Children's Hospital. The database will be anonymised. CRFs will be sent to the study team on paper. This has the added benefit of providing consistency of data entry, allowing for more complete and accurate set of data for long term analysis. This database will use the PUDDLS code only. The data base will be password protected, backed up by the ICH server backup daily, and encrypted for added protection. The database will be accessed according to Trust policies and procedures.

(ii)Healthtracker TM

Healthtracker is hosted with Rackspace on a single server running the Microsoft Windows Server 2003 Standard Edition operating system. Rackspace ensures the server is continuously updated and has all the latest service packs and security patches. The server itself houses a Dual-Core AMD Opteron processor running at 2.20 GHz with 4.0 GB of RAM. It has three 73 GB SCSI hard drives in a RAID 5 configuration offering 140 GB of usable storage. The server acts as both the web and database server running Internet Information Services (IIS) and SQL Server 2005 Standard Edition.

Both the admin and non-admin websites use SSL 128 bit encryption to ensure data is securely encrypted between requests. The websites are written in C# and run under the Microsoft .Net 2.0 framework. HTML and cascading style sheets are used in both sites to apply common styling. All database transactions are performed via stored procedures (there is no in-line SQL). Flash is used to provide some questionnaire and game content to the child portion of the non-admin website. XML web services are used to send and receive information to and from these components. All data in the system is stored in a single SQL Server database and therefore can be exported in any format facilitated by SQL Server. Alternatively custom exports can be written.

Healthtracker (HT) Results are generated in real time and can reflect symptom trends/treatment efficacy/or changes over time. The program has been designed with the ability to analyse large amounts of longitudinal data with inbuilt real-time Bayesian/Neural Net algorithms. This system allows testing of hypothesis as new data comes in. The online presentation of HealthTracker has already meant that rapid acquisition of data for norming, or comparing new measures with existing ones is possible. Non-linear mixed effects modelling will be used to generate estimates of expected recoveries. Data from HT will also be exported via an excel spreadsheet for the research team to analyse. Item analysis of questionnaires will be via Rasch analysis, utilising the WINSTEPS TM statistical software.

• Data protection, security and confidentiality will be implemented according to the UK Data Protection Act 1998 and each Trust's own Information Governance Policy.

## List of abbreviations

ADEM: Acute Disseminated Encephalomyelitis; CNS: Central Nervous System; CIDD: CNS Inflammatory Demyelinating Disease; CRF: Case Report Form; CSF: Cerebrospinal Fluid; EDSS: Expanded Disability Status Scale; Health Tracker^®^: A web-based assessment system which comprises a suite of questionnaires and test games for children. These allow accurate measures of change across a wide range of symptoms, side-effects, psychological functions and quality of life. Assessments can be home-based for convenience and lower costs; HTA: Human Tissue Act 2004 (http://www.opsi.gov.uk/acts/acts2004/ukpga_20040030_en_1); IPMSSG: International Paediatric Multiple Sclerosis Study Group; Life-H: Assessment of Life Habits Questionnaire; MCRN: Medicines for Children Research Network; MRIS: Medical research Information Service; MS: Multiple Sclerosis; MSIS-29: The Multiple Sclerosis Impact Scale; Parent report version; NIHR: National Institute for Health Research; NMO: Neuro-myelitis Optica; ON: Optic Neuritis; PedsQL: Pediatric Quality of Life Inventory TM; PUDDLS: Paediatric UK Demyelinating Disease Longitudinal Study; SDQ: Strengths and Difficulties Questionnaire; SOP: Standard Operating Procedure; TM: Transverse Myelitis; UCID-SS: UK and Ireland Childhood Inflammatory Demyelination Surveillance Study; UKCRN: UK Clinical Research Network; UKCNRC: UK Children's Neurological Research Campaign; Provides neurology network and study management support; WTCRF: Wellcome Trust Clinical Research Facility.

## Competing interests

The authors declare that they have no competing interests.

## Authors' contributions

All authors participated in the design of the study. MA, ML and EW drafted the manuscript. CH, MP, CD, PJ and EW edited the manuscript. All authors read and approved the manuscript.

## Pre-publication history

The pre-publication history for this paper can be accessed here:

http://www.biomedcentral.com/1471-2431/11/68/prepub
